# The use of individual-based FDG-PET volume of interest in predicting conversion from mild cognitive impairment to dementia

**DOI:** 10.1186/s12880-024-01256-x

**Published:** 2024-03-28

**Authors:** Shu-Hua Huang, Wen-Chiu Hsiao, Hsin-I Chang, Mi-Chia Ma, Shih-Wei Hsu, Chen-Chang Lee, Hong-Jie Chen, Ching-Heng Lin, Chi-Wei Huang, Chiung-Chih Chang

**Affiliations:** 1 Department of Nuclear Medicine, Kaohsiung Chang Gung Memorial Hospital, Chang Gung University College of Medicine, Kaohsiung, Taiwan; 2 Department of Neurology, Cognition and Aging Center, Institute for Translational Research in Biomedicine, Kaohsiung Chang Gung Memorial Hospital, Chang Gung University College of Medicine, Kaohsiung, Taiw Taiwan; 3 Department of Statistics, National Cheng Kung University, Tainan City, Taiwan; 4 Department of Radiology, Kaohsiung Chang Gung Memorial Hospital, Chang Gung University College of Medicine, Kaohsiung, Taiwan; 5 Center for Artificial Intelligence in Medicine, Chang Gung Memorial Hospital, Taoyuan, Taiwan; 6 Bachelor Program in Artificial Intelligence, Chang Gung University, Taoyuan, Taiwan

**Keywords:** FDG-PET, MRI, MCI conversion, AD

## Abstract

**Background:**

Based on a longitudinal cohort design, the aim of this study was to investigate whether individual-based ^18^F fluorodeoxyglucose positron emission tomography (^18^F-FDG-PET) regional signals can predict dementia conversion in patients with mild cognitive impairment (MCI).

**Methods:**

We included 44 MCI converters (MCI-C), 38 non-converters (MCI-NC), 42 patients with Alzheimer’s disease with dementia, and 40 cognitively normal controls. Data from annual cognitive measurements, 3D T1 magnetic resonance imaging (MRI) scans, and ^18^F-FDG-PET scans were used for outcome analysis. An individual-based FDG-PET approach was applied using seven volumes of interest (VOIs), Z transformed using a normal FDG-PET template. Hypometabolism was defined as a Z score < -2 of regional standard uptake value ratio. For the longitudinal cognitive test scores, generalized estimating equations were used. A linear mixed-effects model was used to compare the temporal impact of cortical hypometabolism and cortical thickness degeneration.

**Results:**

The clinical follow-up period was 6.6 ± 3.8 years (range 3.1 to 16.0 years). The trend of cognitive decline could differentiate MCI-C from MCI-NC after 3 years of follow-up. In the baseline 18F-FDG-PET scan of the patients with MCI, medial temporal lobe (MTL; 94.7% sensitivity, 80.5% specificity) and posterior cingulate cortex (PCC; 89.5% sensitivity, 73.1% specificity) hypometabolism predicted conversion with high accuracy. ^18^F-FDG-PET hypometabolism preceded dementia conversion at an interval of 3.70 ± 1.68 years and was earlier than volumetric changes, with the exception of the MTL.

**Conclusions:**

Our finding supports the use of individual-based ^18^F-FDG-PET analysis to predict MCI conversion to dementia. Reduced FDG-PET metabolism in the MTL and PCC were strongly associated with future cognitive decline in the MCI-C group. Changes in ^18^F-FDG-PET occurred 1 to 8 years prior to conversion to dementia. Progressive hypometabolism in the PCC, precuneus and lateral temporal lobe, but not MTL, preceded MRI findings at the MCI stage.

**Supplementary Information:**

The online version contains supplementary material available at 10.1186/s12880-024-01256-x.

## Background

The diagnosis of Alzheimer’s disease (AD) includes positive findings of amyloid and tau biomarkers [1]. Currently, AD can be diagnosed at the mild cognitive impairment (MCI) and even cognitively unimpaired stage with positive amyloid and tau biomarkers, however when patients at the MCI stage will convert to dementia is still unclear. One reason for the uncertainty is that MCI now represents a clinical stage between a normal and dementia state, but the term is historically used in a heterogeneous context [2–6]. Differences in case definition may affect the reported rate of MCI conversion, which ranges from 4–60% [7]. Previously, “MCI progression to dementia” and “MCI conversion to AD” were used interchangeably. In 2011, Albert et al. published the core clinical criteria for the diagnosis of MCI due to AD [6]. Using these core clinical criteria, researchers have an operational definition of MCI that aims to control the underlying pathology. Until 2018 [1], MCI stage transition and disease progression belonged to the same research concept in biomarker-validated AD. However, more data are still needed to understand the diagnostic or prognostic value of these criteria.

For MCI conversion, the observation time required for “conversion” is also unknown. Even in AD, the conversion time may not be uniform. Similarly, not all MCI patients convert to dementia. In a 3-year follow-up study in Taiwan, the rate of MCI conversion to dementia was 18.2%/person-years [8]. Whether more conversions from MCI to dementia would occur over a longer follow-up period or at an average time frame requires more data. Vemuri et al. [9] published a time-to-event follow-up study of MCI conversion, in which the conversion duration ranged from 1 to 6 years. Therefore, using a predefined time frame to construct a predictive model of MCI conversion may not be appropriate, since it is likely that those being classified as non-converters will convert at some point in the future.

Despite the high clinical relevance of amyloid and tau biomarkers in the prediction of MCI conversion to AD dementia [10], neuronal injury biomarkers are still of great clinical importance, as they are more accessible and correlated more closely to the clinical features compared with amyloid biomarker alone [11, 12]. Therefore, constructing an individual-based model using neuronal injury biomarkers in MCI is rational. In AD, the combined use of ^18^F fluorodeoxyglucose positron emission tomography (FDG-PET) and magnetic resonance imaging (MRI) has been shown to predict future cognitive decline more precisely than using amyloid scans alone [13, 14]. In addition, evidence has shown that the appropriate use of FDG-PET [15–19] or structural MRI [20] can also achieve a high diagnostic accuracy for AD.

In the past two decades, the use of FDG-PET or structural MRI as an adjuvant to predict dementia conversion has been widely investigated. In FDG-PET, regional hypometabolism of the temporoparietal cortex, posterior cingulate cortex (PCC), and precuneus has been associated with a higher likelihood of dementia conversion [21–28]. For structural MRI, decreased hippocampal or entorhinal volume is the most predictive factor [29–32]. Since most previous studies have used a cross-sectional design, different inclusion criteria, and a relatively short follow-up period, it is possible that MCI conversion did not occur during the observational period or that the results were pointing to different diagnostic entities.

The primary aim of this study was to understand the interval between changes in FDG-PET metabolism within volumes of interest (VOIs) and the occurrence of dementia in a group of patients who met the definition of MCI due to AD according to the core clinical criteria [6].

## Methods

### Standard protocol approval, registration and patient consent

We selected controls, patients with MCI due to AD [6] and patients with AD with dementia [1] from the database of our institute. The Institutional Review Board of our institute approved this study, and written informed consent was obtained from all participants or legally authorized representatives in the cases with cognitive impairment.

### Inclusion and exclusion criteria

Subjects were eligible if they had available brain MRI, FDG-PET, and at least 3 years of clinical follow-up data following FDG-PET. Neuropsychological assessments including the Mini-Mental State Examination (MMSE) [33] Cognitive Ability Screening Instrument (CASI) [34], and Clinical Dementia Rating (CDR) [35] were evaluated annually. The patients with MCI fulfilled the core clinical criteria of MCI due to AD [6], and were further divided into converter (MCI-C) or non-converter (MCI-NC) groups based on their progression to a demented state, as indicated by an MMSE score falling below 20 over a multi-year follow-up period [36]. The MCI group had a mean clinical follow-up period of 6.6 ± 3.8 years (range, 3.1 to 16.0 years). Amyloid-positive AD patients with dementia [1] were also enrolled for statistical comparisons, with the diagnosis confirmed by positive amyloid PET findings which were rated by two independent raters. In the MCI group, 26 out of 82 cases (14 MCI-C, 12 MCI-NC) also underwent amyloid and tau PET at a mean 3.4 years (range, 0–7 years) after the baseline FDG-PET scan. The normal cognitive control group had an MMSE score of > 25, a CASI total score of > 50th percentile, and a CDR score of 0.

The exclusion criteria were lesions on T2-weighted MRI indicating stroke or severe white matter diseases, clinically unmanaged diabetes, or clinical evidence of depression.

### MRI acquisition and preprocessing steps

Three-dimensional (3D) T1 MR images were obtained using a 3T GE Discovery 750 (GE Medical Systems, Milwaukee, WI, USA) and acquired using a T1-weighted, inversion-recovery-prepared, 3D, gradient-recalled acquisition in a steady-state sequence [repetition time (TR) = 12.24 msec; echo time (TE) = 5.18 msec; field of view (FOV) = 256 × 256; matrix size = 256 × 256; number of excitations (NEX) = 1; inversion time (TI) = 450 msec; flip angle = 15] with a 1-mm slice sagittal thickness and a resolution of 0.5 × 0.5 × 1 mm^3^. Details of the preprocessing pipeline are shown in the Supplementary file.

### PET acquisition and preprocessing steps

FDG-PET images were acquired using GE scanners (Discovery ST or MI PET/CT scanner; GE Healthcare, Waukesha, WI). The patients were injected intravenously with 5 mCi FDG after 6 h fasting with a confirmed blood glucose level < 180 mg/dL. After staying in a quiet, dimly room for 30 min, the subjects underwent a 30-minute dynamic PET scan with six 5-minute frames and a low dose CT scan was acquired for attenuation correction. Scans were acquired in 3D mode and reconstructed using an ordered subset expectation maximization algorithm, with 16 subsets and four iterations, yielding a 128 × 128 matrix with a pixel size of 1.56 mm. The images from the dynamic frames were averaged to create a single static image.

### Individualized VOI scale and definition of abnormality in each subject

For individualized FDG-PET VOI analysis, the brain maps were compared with a commercially available normal database, which was normalized to the pons (Cortex ID; GE Healthcare). Seven signet regions were selected using the AAL atlas according to previous studies on FDG-PET brain scans [15–19] to evaluate the predictive values of conversion, including frontal lobe, parietal lobe, medial temporal lobe (MTL), lateral temporal lobe, posterior cingulate cortex (PCC), precuneus, and occipital lobe [37]. Automated voxel-by-voxel Z scores generated by Cortex ID (using age-matched control subjects) were calculated as Z score = [mean database − mean subject]/SD database. A Z score below − 2 was considered to indicate a hypometabolic state for qualitative analysis.

At the individual PET level, standard uptake value ratio (SUVr) images were also calculated using a mid-pons 16 × 16 mm box as the reference region. The FDG-PET images of the patients and controls were normalized to an optimized FDG-PET template [38] using Statistical Parametric Mapping (SPM12)(https://www.fil.ion.ucl.ac.uk/spm/), according to a validated pipeline [39, 40].

### Group analysis of FDG-PET

We conducted an SPM12 analysis of the FDG-PET hypometabolism patterns across the control, MCI-NC, MCI-C and AD groups. FDG-PET images were first co-registered to the corresponding MRI image, and individual MRI images were spatially normalized to the Montreal Neurological Institute template. The spatial normalization parameters were then applied to the corresponding PET image to obtain the final normalized PET image in the Montreal Neurological Institute domain. Group differences in hypometabolism were obtained using voxel-wise analysis. We used PETSurfer (https://surfer.nmr.mgh.harvard.edu/fswiki/PetSurfer) to register a PET scan to its corresponding time point MRI image (Mak et al. 2019). Details of the preprocessing pipeline are shown in the Supplementary file.

### Temporal impact of FDG-PET and MRI

For longitudinal analysis of MRI or PET, we used a linear mixed-effects (LME) model was used with MATLAB 2019b (The Mathworks Inc., Natick, MA, USA) to test the relationships of cortical thickness (or cortical SUVr) with disease duration (months). The temporal impact on cortical hypometabolism and cortical thickness degeneration was assessed, using age, sex and years of education as covariates for both imaging modalities, and additionally total intracranial volume for MRI was also estimated. With cluster-wise correction computed with parametric Gaussian-based simulations to calculate a false positive rate of 0.05, we used a vertex-wise threshold of 3.0 [41].

### Statistical analysis

Statistical analyses were performed in SPSS version 25 (IBM, Armonk, NY), and parameters were described as frequencies for categorical variables and mean ± standard deviation for continuous variables. Differences among the four diagnostic groups (AD, MCI-C, MCI-NC, controls) were assessed using the chi-square test for categorical variables and Kruskal-Wallis test with Bonferroni correction for continuous variables. The relationships between continuous variables were explored using Spearman’s correlation analysis. Sensitivity and specificity were calculated for each subject’s individual VOIs with hypometabolism to predict conversion to dementia. We conducted a one-sample Kolmogorov-Smirnov test to assess the normality of FDG-PET Z scores, MMSE, and CASI scores. As there was no evidence suggesting that FDG-PET Z scores did not follow a normal distribution, we employed a linear mixed-effects (LME) model to conduct longitudinal analysis of the FDG-PET imaging biomarker. Besides, we conducted repeated-measures analysis to assess the differences in VOI metabolism for patients who underwent two FDG-PET scans, using the Wilcoxon Signed Ranks test. Furthermore, the results of the Kolmogorov-Smirnov test indicated that MMSE and CASI scores did not conform to normal distributions. For the longitudinal MMSE and CASI data, we employed a Generalized Estimating Equation (GEE) model. This model incorporated covariates such as gender, age, education years, groups, measurement numbers, and interactions between groups and measurement numbers. Variables were retrieved from demographic data and significant FDG-PET imaging biomarkers. Results were considered significant at *p* < 0.05.

## Results

### Demographic data and FDG-PET

**Fig. 1:**
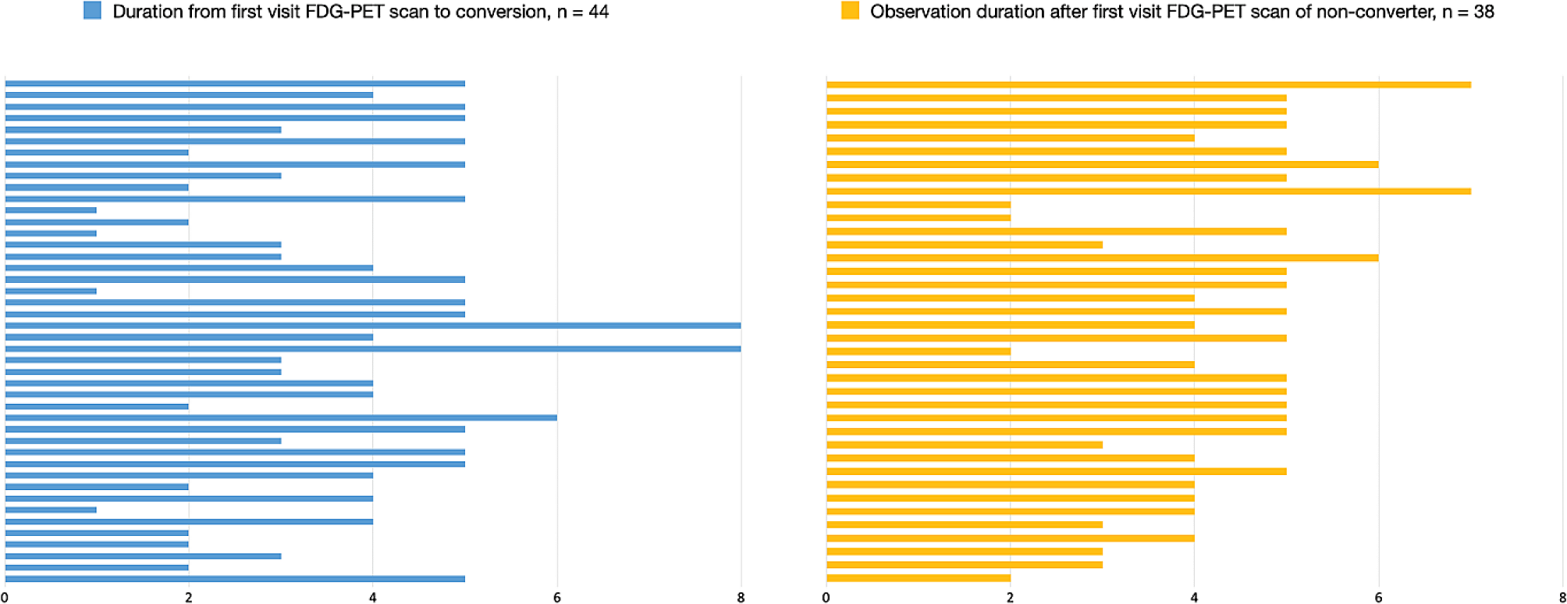
Intervals of MCI conversion to dementia after the baseline FDG-PET scan (**A**) and observation intervals of MCI non-converter after the baseline FDG-PET scan (**B**). Bars represent the follow-up years of each case. Abbreviations: MCI, mild cognitive impairment; AD, Alzheimer’s disease

A total of 164 subjects were enrolled, including 44 MCI converters (MCI-C), 38 non-converters (MCI-NC), 42 patients with AD dementia, and 40 cognitively normal controls, all of whom had baseline FDG-PET scan (Table 1). Among the MCI group, 37 patients received two or more FDG-PET scans (MCI-C: 24, MCI-NC: 13). In the control group, only baseline FDG-PET scans were conducted, and there were no follow-up FDG-PET scans performed. The time interval from the baseline FDG-PET scan to the diagnosis of dementia in the MCI-C group and the observation intervals in the MCI-NC group are shown in Fig. 1. The average time between the baseline FDG-PET scan and conversion to dementia was 3.70 ± 1.68 years (range, 1 year to 8 years). The average follow up time after the baseline FDG-PET scan in the MCI-NC group was 4.34 ± 1.26 years (range, 2 years to 7 years).

At the baseline FDG-PET scan, there were no significant differences in mean age, sex, education, and baseline MMSE score between the two MCI groups (*p* = 0.156, 0.234, 0.187, and 0.579). However, there were significantly fewer *APOE* Ɛ4 carriers in the MCI-C group (*p* < 0.05). There was a significant difference in baseline MMSE score between the MCI and AD groups (*p* < 0.05), and the cognitive test scores were lowest in the patients with AD.

**Table 1:** Demographic data of four groups

	Control	MCI		AD
		Non-converter	Converter	
Sample size	40	38	44	42
Age at baseline FDG-PET	62.6 ± 11.6	72.1 ± 7.60*	74.7 ± 7.04*	77.0 ± 7.6*
Gender (male/female)	23/17	15/23*	19/25*	21/21*
Education (years)	13.6 ± 3.7	6.9 ± 5.0*	7.1 ± 4.9*	7.5 ± 5.3*
*APOE* Ɛ4 carriers	23.8%	37.8%*	60.9%*^§^	57.9%*
Baseline MMSE	28.1 ± 2.1	23.5 ± 4.36*	21.8 ± 3.7*	17.4 ± 2.9*^§#^
Baseline Cognitive ability Screening Instrument				
Total scores (100)	92.6 ± 6.1	77.84 ± 13.9*	74.9 ± 12.2*	63.1 ± 15.3*^§#^
Long term memory (10)	9.88 ± 0.7	9.2 ± 1.8*	9.1 ± 2.6*	7.9 ± 3.6*^§#^
Short term memory (12)	10.5 ± 1.7	7.1 ± 3.6*	4.1 ± 4.0* ^§^	3.8 ± 1.5*^§#^
Attention (8)	7.3 ± 0.8	6.8 ± 1.2*	6.7 ± 1.4*	6.5 ± 1.3*^§#^
Mental manipulation (10)	8.8 ± 1.8	6.3 ± 2.9*	7.0 ± 3.2*	6.3 ± 2.5*^§#^
Orientation (18)	17.7 ± 1.0	15.0 ± 4.0*	11.7 ± 5.1* ^§^	10.9 ± 2.6*^§#^
Drawing (10)	9.8 ± 0.5	8.3 ± 2.3*	8.1 ± 2.9*	7.2 ± 3.6*^§#^
Abstract thinking (12)	10.8 ± 1.2	8.5 ± 2.3*	8.3 ± 2.7*	7.5 ± 2.7*^§#^
Verbal fluency (10)	8.2 ± 2.1	5.7 ± 2.3*	5.8 ± 2.8*	4.8 ± 1.8*^§#^
Language (10)	9.8 ± 0.8	9.0 ± 1.5*	8.7 ± 2.1*	4.9 ± 2.1*^§#^
Baseline FDG-PET SUVr				
Frontal lobe	1.41 ± 0.10	1.45 ± 0.04	1.35 ± 0.06* ^§^	1.30 ± 0.07*^§#^
Medial temporal lobe	1.10 ± 0.07	1.06 ± 0.05	0.91 ± 0.07 *^§^	0.86 ± 0.02*^§#^
Lateral temporal lobe	1.40 ± 0.12	1.38 ± 0.04	1.23 ± 0.08* ^§^	1.19 ± 0.11*^§#^
Posterior cingulate cortex	1.61 ± 0.13	1.60 ± 0.20	1.37 ± 0.14 *^§^	1.20 ± 0.07*^§#^
Precuneus	1.61 ± 0.12	1.61 ± 0.05	1.48 ± 0.09* ^§^	1.31 ± 0.08*^§#^
Parietal lobe	1.50 ± 0.10	1.49 ± 0.04	1.36 ± 0.08* ^§^	1.21 ± 0.06*^§#^
Occipital lobe	1.60 ± 0.11	1.59 ± 0.05	1.56 ± 0.08	1.39 ± 0.12*^§#^
Follow-up (years)	4.4 ± 1.8	6.4 ± 3.8*	6.7 ± 3.6*	6.6 ± 3.2*

Data presented as mean ± standard deviation

* *p* < 0.05 with control; ^§^*p* < 0.05 with MCI non-converter; ^#^*p* < 0.05 with MCI converter

Abbreviations: MCI, mild cognitive impairment; AD, Alzheimer’s disease; *APOE*, Apolipoprotein E; MMSE, mini-mental state examination; FDG-PET, fluorodeoxyglucose positron emission tomography; SUVr, standard uptake value ratio

### Individual analysis of baseline FDG-PET

In the baseline FDG-PET scan, the SUVrs of the preselected VOIs, except for the occipital lobe, were significantly lower in the MCI-C group (*p* < 0.05). For the MCI-C group, the percentages of hypometabolism in the PCC, MTL, precuneus, frontal, parietal, and lateral temporal lobe were 75.2%, 70.0%, 41.2%, 40.0%, 36.7%, and 32.0%, respectively, compared to 12.8%, 5.2%, 8.1%, 5.0%, 5.2%, and 5.0% in the MCI-NC group (Supplementary Fig. 1).

In the baseline FDG-PET scan of the two MCI groups, MTL hypometabolism predicted future conversion with 94.7% sensitivity and 80.5% specificity, followed by the PCC (89.5% sensitivity and 73.1% specificity). Thirty-four cases of the 82 MCI patients were categorized as having normal metabolism status in MTL and PCC areas; all of these patients were in the MCI-NC group. Of note, three patients with MCI showed MTL and PCC hypometabolism but remained cognitively stable (no-conversion) during follow-up.

### Correlation between baseline FDG-PET and MMSE scores

**Fig. 2:**
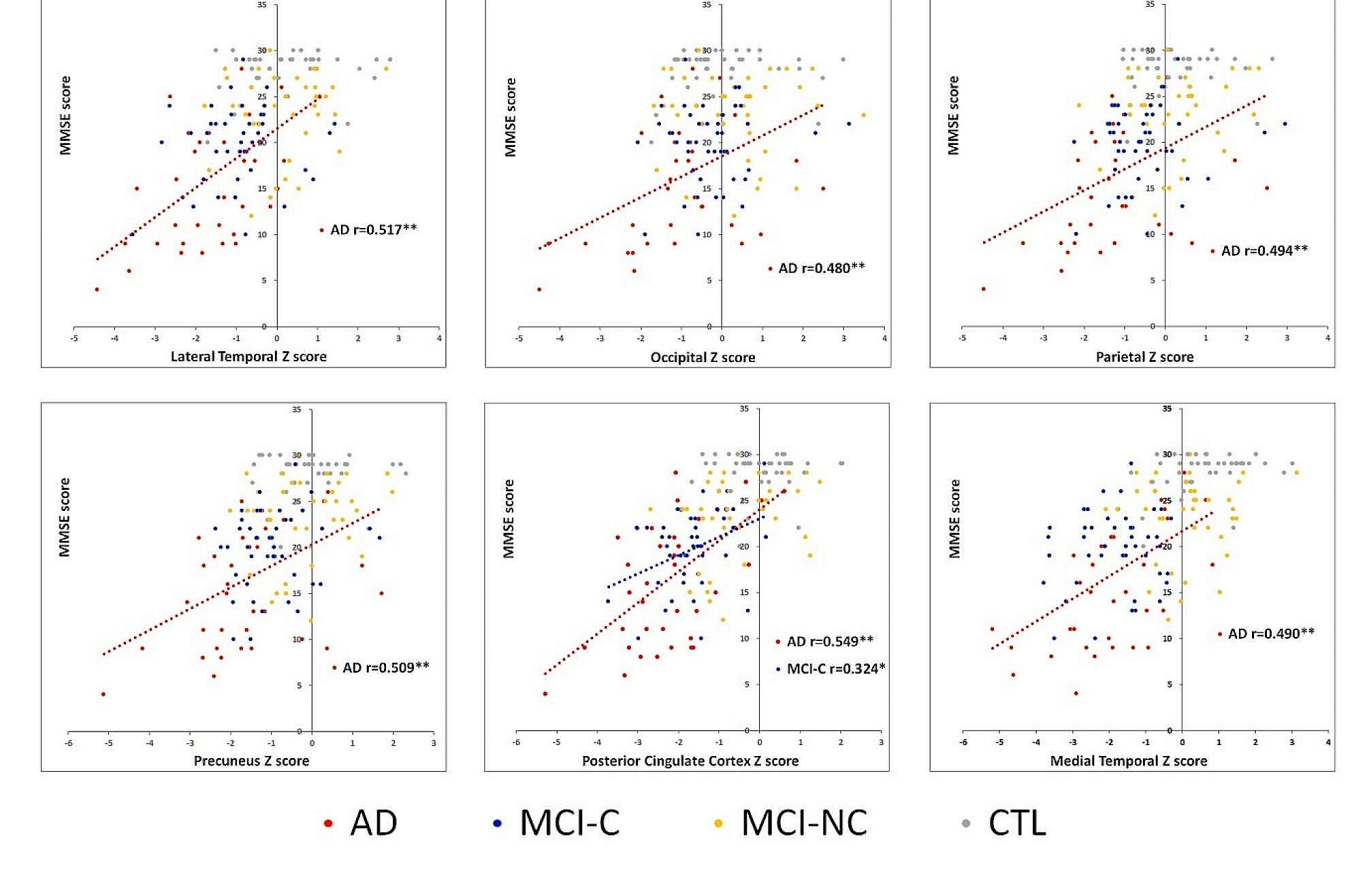
Scatter plot of the correlations between the baseline FDG-PET Z scores from six volumes of interest and baseline MMSE scores. Dots represent each participant in this study; the significant linear relationships are shown in dot lines. r = correlation coefficient; * *p* < 0.05; ** *p* < 0.01. Abbreviations: MCI-NC, non-converter of mild cognitive impairment; MCI-C, converter of mild cognitive impairment; AD, Alzheimer’s disease; MMSE, mini-mental state examination

The correlation between baseline FDG-PET Z scores and corresponding baseline MMSE scores, best fit in the AD group (Fig. 2). Among the preselected VOIs, six had significant correlations with MMSE scores in the AD group. While in the MCI-C and MCI-NC groups, there were no significant correlations between FDG-PET Z scores and corresponding MMSE scores except for the PCC region.

The same analysis was conducted with the CASI and its subdomains to clarify whether hypometabolism in different brain regions was associated with cognitive impairment of different domains (Supplementary results and Supplementary Table 1).

### A representative case with three consecutive FDG-PET scans

**Fig. 3:**
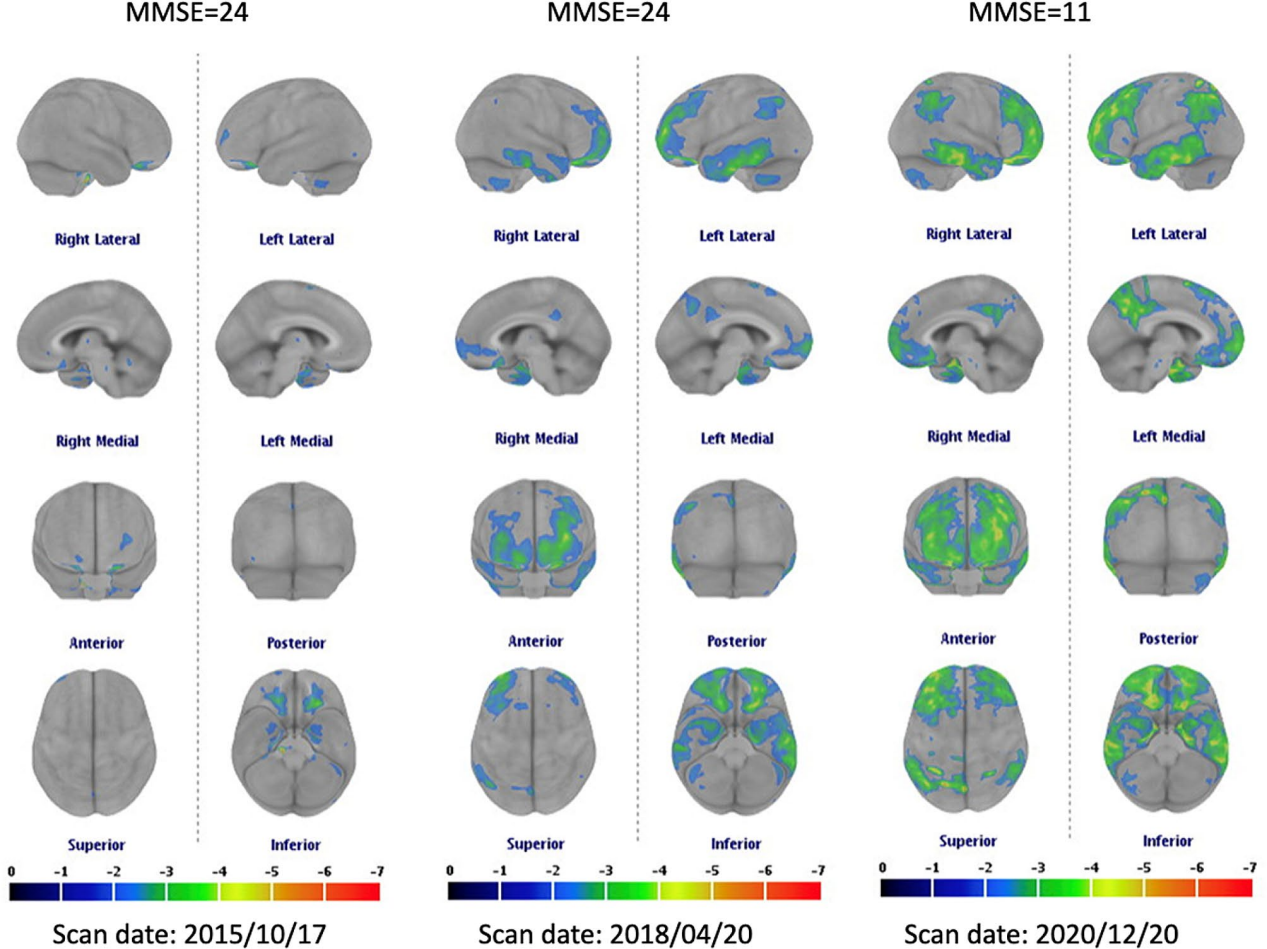
FDG-PET Z score map in a female patient showed conversion in three scans. At the baseline, decreased uptake was noted in the medial temporal lobe and inferior frontal cortex (MMSE = 24; 4 years before dementia). 2.5 years later, hypometabolism was noted in the lateral temporal lobe, posterior cingulate cortex and dorsolateral prefrontal cortex (MMSE = 24; 1.5 years before dementia). Five years after the baseline FDG-PET scan, hypometabolism was more widely spread with a pattern similar to the default mode network (MMSE = 11). Only regions showing a Z score < -2 are displayed. Abbreviations: MMSE, mini-mental state examination

A representative example of a patient with MCI-C depicting the evolutional pattern of three consecutive FDG-PET scans is shown in Fig. 3. Hypometabolism (Z score < -2) started in the MTL and inferior frontal region, followed by lateral temporal, PCC, parietal and frontal cortex regions years before the cognitive changes.

### Longitudinal cognitive trajectory

**Fig. 4:**
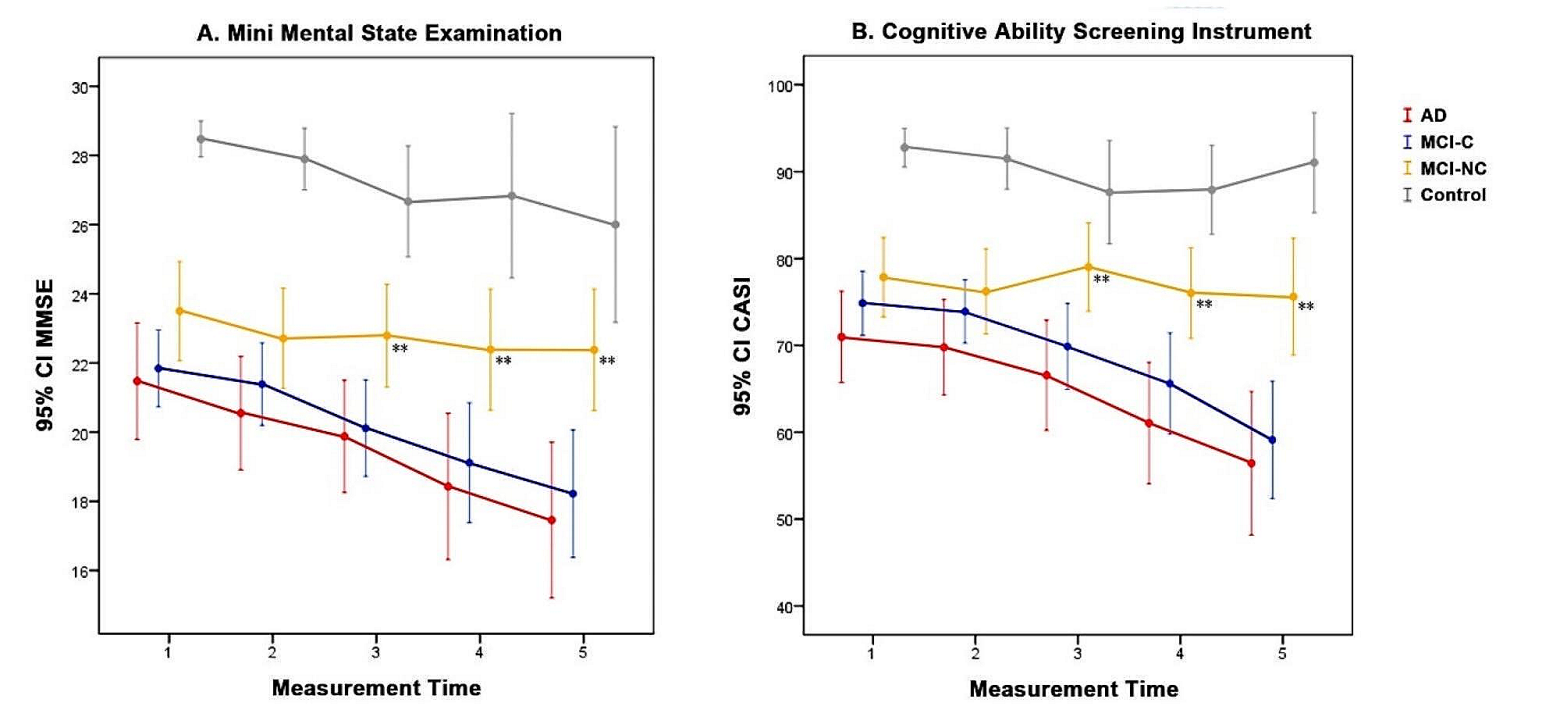
Cognitive test scores of the five consecutive MMSE (**A**) and CASI (**B**) measurements. The progression trajectories of the MCI-C group were similar to the AD group. In contrast, the declining trend of the MCI-NC group was not as conspicuous as the AD or MCI-C group. The difference between the MCI-C and MCI-NC groups became significant from the third MMSE and CASI measurements. The average interval between each measurement was 1 year. ** indicates *p* < 0.05 comparing the MCI-C and MCI-NC groups. Abbreviations: MCI-NC, non-converter of mild cognitive impairment; MCI-C, converter of mild cognitive impairment; AD, Alzheimer’s disease; MMSE, Mini-Mental State Examination; CASI: Cognitive Ability Screening Instrument total scores

Based on the median follow-up duration, five consecutive measurements of MMSE and CASI total scores in the four groups are shown in Fig. 4. There was an approximate interval of 1 year between each measurement. The MCI-C and AD groups had similar progression trajectories, showing a continuous decline in MMSE and CASI scores as the number of measurements increased. In contrast, the trends of cognitive decline in the MCI-NC and control groups were less obvious. There were no significant differences in MMSE and CASI total scores between the MCI-C and MCI-NC groups in the first (*p* = 0.059; 0.110) and second (*p* = 0.116; 0.350) measurements, however the difference was significant in the third measurement (*p* < 0.05). There were interactions between the groups and the number of MMSE and CASI measurements. As a result, the temporal impact was modeled by dividing the data into two stages: (1) data within the first 2-year follow-up period representing the initial stage of the disease; and (2) data pooled from the entire follow-up period (mean: 6.6 ± 3.8 years) representing the disease progression stage. Data of the cognitive features of the two MCI groups are listed in the Supplementary results and Supplementary Table 2.

### Group analysis

Analysis of FDG-PET hypometabolism patterns in the control, MCI-NC, MCI-C and AD groups is shown in the Supplementary Fig. 2. The results suggested that only PCC hypometabolism was detected in the MCI-NC group, while there was a greater spatial extent of hypometabolism in precuneus, PCC, MTL, and temporo-parietal cortices in the MCI-C and AD groups compared with the controls. The regions of hypometabolism detected in the PCC, MTL cortices in the MCI-C group compared with the MCI-NC group were in consistent with individual-based FDG-PET abnormalities.

**Fig. 5:**
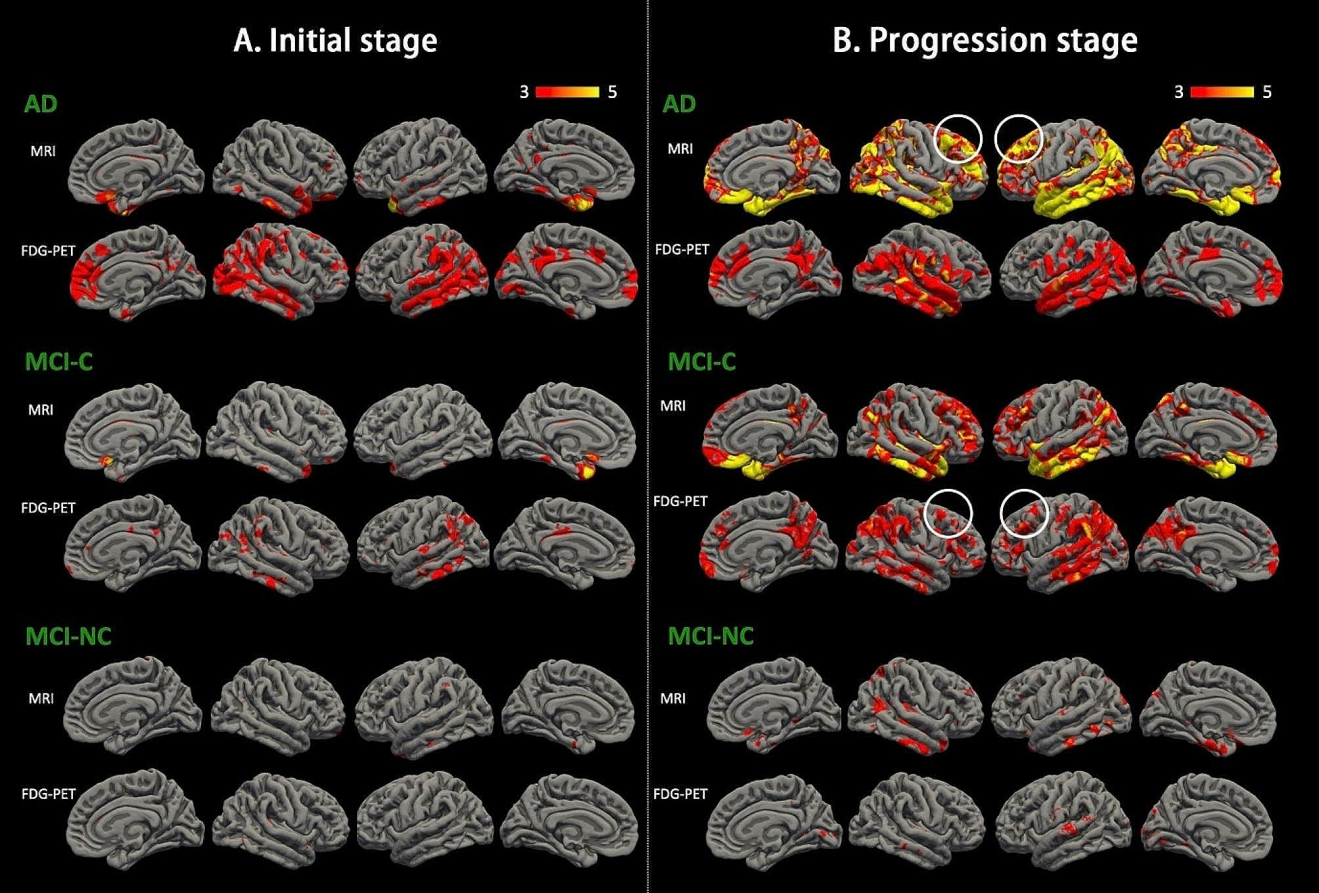
Temporal impact of cortical hypometabolism (FDG-PET) and cortical thickness degeneration (volumetric MRI) in the AD, MCI-C and MCI-NC groups. (**A**) Data from the first 2 years of follow-up represents the initial stage of disease. (**B**) Pooled data from the entire follow-up period (mean: 6.6 ± 3.8 years) represents the disease progression stage. The circles indicate the dorsolateral prefrontal cortex. Significance was set at a vertex-wise threshold of 3.0. Abbreviation: MCI-NC, non-converter of mild cognitive impairment; MCI-C, converter of mild cognitive impairment; AD, Alzheimer’s disease; MRI, magnetic resonance imaging

Figure 5 shows group-level FDG-PET hypometabolism across disease stage and cortical thickness degeneration in the MCI-C, MCI-NC and AD groups. According to the predefined time frame, the temporal impact of FDG-PET hypometabolism and MRI cortical thickness degeneration was divided into the initial stage of disease (first 2 years of follow-up) (Fig. 5A), and disease progression stage (the entire follow-up period) (Fig. 5B). The FDG-PET hypometabolic temporal impact in the AD group suggested a greater spatial extent of hypometabolism (over the medial prefrontal, lateral temporal, temporal-parietal and precuneus regions; Fig. 5A) in the initial stage of disease compared to the corresponding cortical thickness degenerative trajectory (only over the hippocampus). As the disease progressed, the pattern of cortical thickness degenerative became roughly overlapped with that of hypometabolism. However, distinct patterns persisted in the MTL, precuneus, and a large part of the superior frontal lobe, where atrophy was observed but no hypometabolism in the FDG-PET AD-stage (Fig. 5B). In the MCI-C group, the evolutional pattern of hypometabolism in the initial stage of disease (Fig. 5A) was similar to the AD group, but with a relatively sparse distribution in the PCC, precuneus and lateral temporal lobe. As the disease progressed, the hypometabolism pattern mimicked that of AD (Fig. 5B). One exception was the temporal impact on the dorsolateral prefrontal cortex (DLFC), which showed progressive hypometabolic changes in the MCI-C group (observed at the disease progression stage) but not in the AD group (in either the initial stage or progression stage); instead, in the AD group, cortical atrophy over the DLFC was observed. Hypometabolism or cortical atrophy in the MCI-NC group was inconspicuous at the initial stage; however, as the follow-up duration increased, cortical atrophy over the hippocampus and lateral temporal lobe was noted. Of note, there was no signal change in the hippocampus in the FDG-PET temporal impact model at either the initial stage or disease progression stage among the three groups. In fact, MTL hypometabolism, defined as a Z score < -2, was noted in the baseline FDG-PET scan in both the MCI-C (70%) and AD (81%) groups.

### Distinct FDG-PET topographies of MCI converters and non-converters

**Fig. 6:**
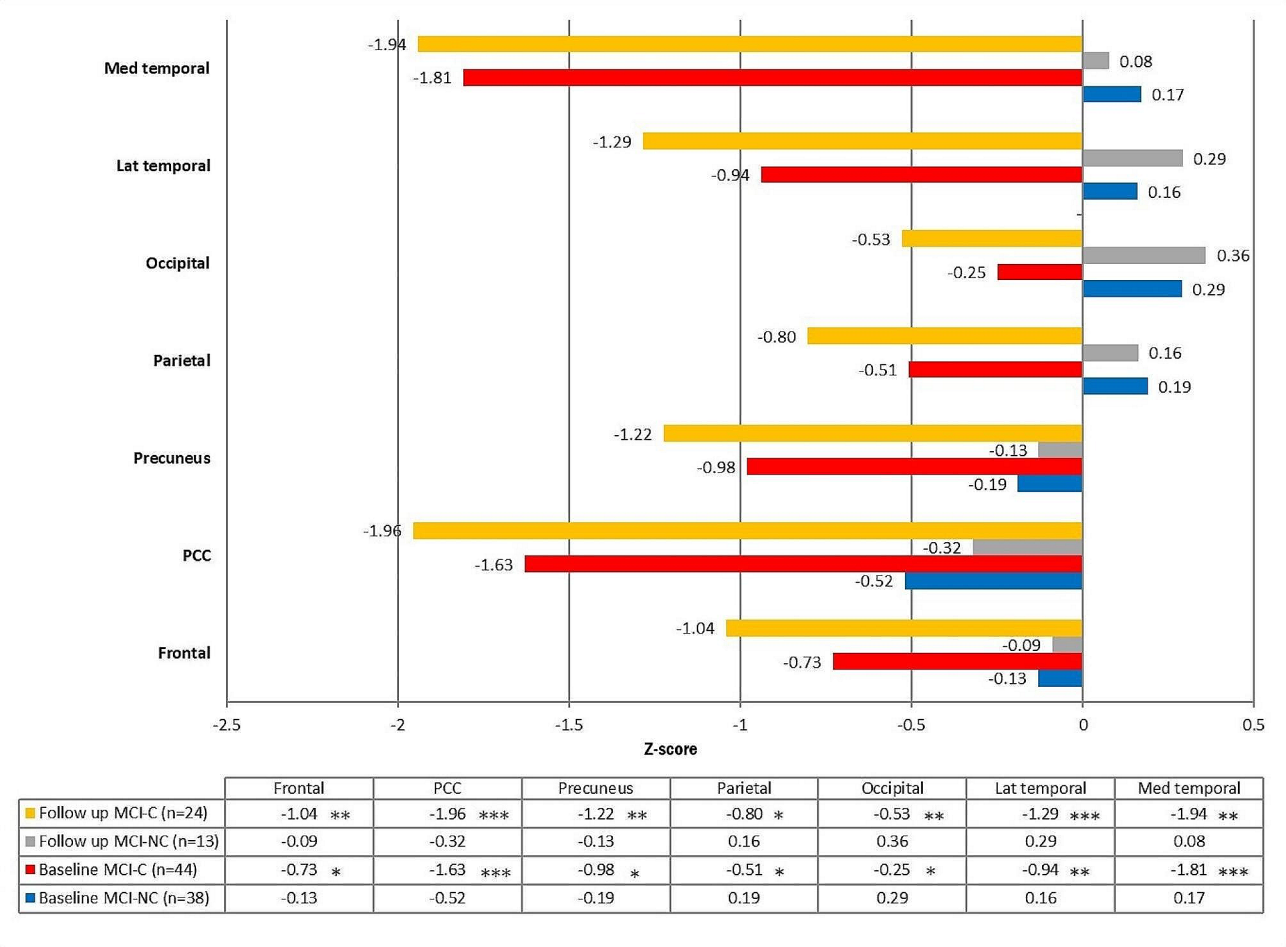
FDG-PET topography of the preselected seven volumes-of-interests. The MCI-C group showed prominent hypometabolism in the medial temporal lobe and PCC, and the FDG-PET uptakes over all regions decreased in the baseline and follow-up scans. The difference in the baseline scan became more robust in the follow-up scan. In contrast, the MCI-NC group seemed to have a relatively hypometabolic change in the PCC in the baseline scan; however, this was not noted in the follow-up scan, and there was no significant declining trend over all regions. Numbers on the chart indicate the Z score of FDG-PET. Using Mann-Whitney U test to compare between MCI-C and MCI-NC; * *p* < 0.05; ** *p* < 0.01; *** *p* < 0.001. Abbreviation: MCI-NC, non-converter of mild cognitive impairment; MCI-C, converter of mild cognitive impairment; PCC, posterior cingulate cortex; Med. temporal, medial temporal; Lat. temporal, lateral temporal

Repeated-measures analysis was conducted in the patients with MCI who received two FDG-PET scans (MCI-C: 24, MCI-NC:13), and the preselected seven VOIs between the two MCI groups and the two scans were compared (Fig. 6). There was no significant difference in the mean age at the two FDG-PET scans between the two groups. In the baseline scan, the MCI-C group had significantly lower frontal, PCC, precuneus, lateral temporal, and MTL Z scores. During follow-up, the Z scores of all VOIs became significantly lower in the MCI-C group. The difference could be observed from the baseline FDG scan and became more evident during follow-up (Supplementary Table 3).

### Generalized estimating equation model revealed the factors correlated with cognitive decline

To better understand which factors affected cognitive change, a generalized estimating equation model was used for the longitudinal data. As shown in Table 2, the FDG-PET PCC, precuneus and lateral temporal lobe Z scores were positively correlated with MMSE scores, of which the lateral temporal lobe had the most significant effect. In contrast, the Z scores of the frontal lobe had an inverse effect on the MMSE scores. Similar findings were seen when using CASI total scores as the dependent variable.

**Table 2:** The coefficients of covariates in the generalized estimating equation model for MMSE and CASI scores

		MMSE			CASI		
		*β*	SE	*p* value	*β*	SE	*p* value
Male gender		1.198	0	**0.025** ^*^	4.694	1.901	**0.014** ^*^
Education year		0.415	0.074	**< 0.001** ^***^	1.376	0.252	**< 0.001** ^***^
FDG-PET Z score							
	Frontal	-2.289	0.496	**< 0.001** ^***^	-9.620	1.616	**< 0.001** ^***^
	PCC	1.637	0.497	**0.001** ^**^	4.676	1.794	**0.009** ^**^
	Precuneus	1.434	0.657	**0.029** ^*^	4.867	2.315	**0.036** ^*^
	Parietal	-0.968	0.660	0.143	-1.729	2.519	0.492
	Occipital	-0.244	0.587	0.667	-1.582	1.759	0.368
	Lat. temporal	1.891	0.547	**0.001** ^**^	8.733	1.938	**< 0.001** ^***^
	Med. temporal	-0.011	0.266	0.967	-0.574	0.899	0.523
Measurement number, AD							
	2nd time	-0.847	0.632	0.180	-1.573	1.761	0.372
	3rd time	-1.020	0.765	0.182	-3.177	2.242	0.157
	4th time	-2.256	1.123	**0.045** ^*^	-8.341	3.017	**0.006** ^**^
	5th time	-3.004	1.227	**0.014** ^*^	-15.092	3.618	**< 0.001** ^***^
	6th time	-5.325	2.154	**0.013** ^*^	-21.842	4.378	**< 0.001** ^***^
Measurement number, MCI-C							
	2nd time	-0.339	0.590	0.565	-1.260	1.637	0.441
	3rd time	-0.888	0.719	0.217	-2.628	2.045	0.199
	4th time	-1.990	0.999	**0.046** ^*^	-7.282	2.780	**0.009** ^**^
	5th time	-2.730	1.074	**0.011** ^*^	-15.957	3.322	**< 0.001** ^***^
	6th time	-5.116	2.097	**0.015** ^*^	-22.565	4.022	**< 0.001** ^***^
Measurement number, MCI-NC							
	2nd time	-0.687	0.633	0.278	-1.890	2.016	0.348
	3rd time	0.557	0.734	0.448	4.240	2.059	**0.039** ^*^
	4th time	-0.076	0.912	0.933	0.248	2.375	0.917
	5th time	-0.237	0.942	0.802	-2.682	3.169	0.397
	6th time	-1.206	2.098	0.566	-8.594	3.983	**0.031** ^*^

Abbreviations: MCI-NC, non-converter of mild cognitive impairment; MCI-C, converter of mild cognitive impairment; AD, Alzheimer’s disease; MMSE, mini-mental state examination; CASI, cognitive ability screening instrument total score; FDG-PET, fluorodeoxyglucose positron emission tomography; PCC, posterior cingulate cortex; Lat. temporal, lateral temporal; Med. temporal, medial temporal; SE, standard error

* *p* < 0.05; ** *p* < 0.01; *** *p* < 0.001

Furthermore, using time as a categorical variable, we found negative interactions between the number of measurements and cognitive test scores in the AD and MCI-C groups but not in the MCI-NC group. In addition, the interactions became significant after the third measurement. Interestingly, the MCI-NC group showed some improvement in cognitive test performance during follow-up; for example, the third MMSE scores increased by 0.557 points from baseline, and the third CASI total scores increased by 4.204 points. Nevertheless, the performance worsened from the fourth measurement. The trends in cognitive decline were most prominent in the AD and MCI-C groups.

### MCI patients with amyloid and tau PET

In the 26 MCI patients who also underwent amyloid and tau PET in the following mean 3.4 years (range, 0–7 years) after the baseline FDG-PET scan, 41.7% (5 out of 12) of those with MCI-NC and 92.9% (13 out of 14) of those with MCI-C were found to have significant Alzheimer’s pathology based on both amyloid and tau PET positivity. Details of the amyloid positive/negative and tau positive/negative analyses in the MCI patients are shown in the Supplementary file.

## Discussion

### Major findings

In this study, we assessed longitudinal neuropsychological tests (MMSE and CASI), FDG-PET and structural MRI in subjects with MCI-C and MCI-NC, and constructed cognitive, cortical hypometabolism, and cortical degeneration features to predict conversion. There were three significant findings. First, the applicable model to predict MCI conversion was based on the individual-based VOI approach using one FDG-PET scan at the MCI stage. Hypometabolism of the MTL and PCC (Z score <-2) reached the highest specificity at the subject level, while the predictive abnormalities could be detected at 3.70 ± 1.68 years prior to the occurrence of dementia, although we also observed an 8-year interval between the FDG-PET scan showing MTL hypometabolism and the conversion to dementia. Second, we assessed the relationships between the evolution of two neuronal injury biomarkers with related cognitive decline patterns. We found that the patients with MCI-C and AD followed similar degenerative trajectories, supporting the clinical significance of the core clinical criteria of MCI due to AD [6] with the neuronal injury biomarkers in the MCI predictive model. Finally, we explored longitudinal data, and found that progressive decrease in glucose metabolism over the PCC, precuneus and lateral temporal lobe could reflect cognitive decline. Moreover, comparing cortical hypometabolism and cortical atrophy at different disease stages suggested that FDG-PET had higher sensitivity in detecting regional metabolic abnormalities.

### FDG-PET as a biomarker of MCI conversion

Both FDG-PET and MRI represent neuronal injury biomarkers in AD [11]. Hypometabolism of temporoparietal and PCC regions in FDG-PET has been shown to be a typical feature of AD [42], and it has been reported as early as the preclinical or prodromal stage [43]. To predict MCI conversion using FDG-PET, hypometabolism over the PCC, precuneus or temporoparietal cortex has frequently been reported [21, 23–27, 44–53]. However, the application of a subject-level-based VOI approach in MCI conversion is novel. We found that changes in the PCC could predict conversion, however, changes in the PCC may have been less specific than those in the MTL based on the relatively higher proportion of hypometabolism in the PCC compared to the MTL (12.8% vs. 5.2%) in the MCI-NC group. The role of the MTL as a predictor for MCI conversion has rarely been reported; this may be related to the temporal course of the disease, as the evolutional pattern of glucose metabolic abnormalities has been shown to occur relatively early in the hippocampus compared to the temporoparietal cortex and PCC in MCI [54]. This evolutional pattern was also observed in our FDG-PET temporal impact analysis; as illustrated in Fig. 5A, the temporal impact of SUVr change over the hippocampus was limited compared to the PCC, even at the initial stage of MCI-C or AD. We propose that hypometabolism over the MTL occurs at the very early stage of disease. Since our follow-up duration was longer than in most previous studies, the hypometabolism in the MTL may be an earlier indicator of MCI conversion than hypometabolism in the PCC.

Moreover, an important aspect of FDG-PET in MCI is its role of exclusion. In our series, 34 MCI patients did not demonstrate hypometabolism in FDG-PET and 33 did not show progression. The high specificity (93.7%) and negative predictive value (93.7%) can be extremely useful to rule out progression in MCI. A normal FDG-PET scan at the MCI stage has been shown to be a reliable indicator of non-progression or for reconsidering the diagnosis of neurodegenerative disease [55, 56].

### Cognitive decline trajectory still reflected MCI conversion at year 3

Although previous studies on the use of neuropsychological tests to predict MCI conversion have commonly focused on the predictive value of single memory tests [57, 58], our outcome measures focused on the longitudinal changes and interval to conversion to dementia. We found similar declining trends of MMSE and CASI scores in the MCI-C and AD groups, but not in the MCI-NC group. In addition, the differences became significant after the third year of follow-up. As both patients with MCI-C and MCI-NC were enrolled based on the MCI due to AD criteria, the declining trend in MCI-C suggests that at least 3 years of follow-up is required for patients with MCI to predict the conversion to dementia. Based on our finding that the baseline FDG-PET hypometabolism could predict conversion with high accuracy, we propose that the hypometabolism begins at least 3 years before the clinical onset of dementia. The declining patterns in the MCI-C and AD groups were similar, meaning that the longitudinal follow-up of MCI could still reveal possible neuronal injury similar to AD.

### Multi-modal model for MCI conversion

Structural MRI is considered to be of equal value to FDG-PET as a neuronal injury biomarker [1]. The use of MRI in assisting the prediction of MCI conversion has also been widely investigated [29–32, 59–62]. The issue of whether MRI is superior to FDG-PET is still under debate, which could be related to the variety of selected metrics when evaluating the two imaging modalities and the study populations [63]. In our temporal impact analysis of the two imaging modalities, FDG-PET revealed more widespread abnormalities in the PCC, precuneus and lateral temporal lobe, but not the MTL, at the initial stage of MCI compared with MRI. This pattern is consistent with the typical temporal sequence of biomarker changes in AD [11]. Meanwhile, we also observed that group FDG-PET signal reduction reached a plateau in both the MCI-C and AD groups at the initial stage of disease. In contrast, MRI abnormalities were restricted mainly to the hippocampus at the initial stage; however, with disease progression, more extended atrophy was noted compared to the temporal impact on FDG-PET. In line with other studies [49, 64], we suggest that progressive hypometabolism in the PCC, precuneus and lateral temporal lobe, and progressive cortical thickness degeneration in the hippocampus in the early disease stage of MCI; whereas in the late stage of MCI, MRI has an important role in monitoring disease progression. Since there is currently no standard definition of the severity of MCI, and as the enrolled time point of MCI patients would differ among different studies, it is very likely that the enrolled MCI patient would have different stages, therefore resulting in inconsistent findings across different studies. With advances in the diagnosis and interventions for AD at the prodromal stage, constructing a uniform scale to evaluate the stage or severity of MCI could be of clinical and research importance.

### Evolution in FDG-PET reflected cognitive decline

Instead of hypometabolism, increased Z scores over the frontal lobe were found in the patients with cognitive decline in our longitudinal data (Table 2). In early AD, activation over the DLFC and parietal-temporal border has been reported to be higher than in the general population in FDG-PET when performing verbal episodic memory tasks [65]. Similarly, in an MCI functional MRI study, increased activation over frontal regions was found when performing memory-related tasks [66]. We speculate that the progressively increases in frontal FDG-PET signal could be a compensatory mechanism during the loss of cognitive function. This aligns with previous studies demonstrating that compensatory hypermetabolism can manifest in MCI subjects, where the elevated metabolic rate observed across various cortical regions might represent a compensatory mechanism in response to early neuronal damage in the progression of AD [67]. As shown in Fig. 5, we found cortical hypometabolism over the DLFC in the disease progression stage but not in the initial stage of MCI-C, and cortical atrophy over the DLFC in the patients with AD. This suggests that failure of DLFC compensation could be an indicator of AD conversion.

### Restricted pathology status in the MCI patients

Due to the extended duration of this longitudinal study, there were constraints on conducting pathological examinations in the early period. As a result, only 26 MCI patients underwent amyloid and tau PET examinations following FDG-PET. The patients with MCI-C exhibited a higher frequency of amyloid and tau PET positivity compared to those with MCI-NC. Notably, 41.7% (5 out of 12) of the patients with MCI-NC and 92.9% (13 out of 14) of those with MCI-C were found to have both amyloid and tau PET positivity. This implies that a majority of the MCI-C patients in this study exhibited AD pathophysiology, and that a minor subset of the MCI-NC patients also demonstrated positive AD pathology. Further follow-up of these MCI-NC patients and the development of an integrated biomarker model to assess amyloid and tau PET examinations, along with FDG-PET, for the early detection of MCI conversion to dementia, could hold clinical significance [68, 69].

### Limitations and strength

This study had two limitations. First, the underlying neuropathology of MCI was not assessed in all cases; therefore, the definite diagnosis of MCI could still be heterogeneous. As our MCI patients were enrolled based on the core clinical criteria of MCI due to AD, and as the MCI-C patients showed a similar degenerative trajectory to the AD patients. It is highly likely that a significant proportion of the MCI-C group exhibited AD pathophysiology, which is consistent with our constrained findings from AD pathological examinations. Second, because this was a retrospective analysis, not all of the patients with MCI underwent a second FDG-PET scan, and the interval between two scans was not standardized. However, the randomly scattered time points of FDG-PET with an individualized prediction model may enhance its clinical application. For the follow-up FDG-PET scans, we used a GEE model to evaluate the longitudinal changes in SUVr signals, and their correlations with cognitive decline were not significant. This suggests that cross-sectional FDG-PET was adequate to build the model, and that the non-fixed timing of FDG-PET scans could be considered to reflect real-world conditions.

## Conclusion

In conclusion, our results showed that FDG-PET tailored to individual patients with MCI with a VOI approach could assist in the prediction of future progression. Reduced FDG-PET metabolism in the MTL and PCC were strongly associated with future cognitive decline in the MCI-C group. The average interval between changes in FDG-PET signal and dementia conversion was 3 years (range, 1 to 8 years). Progressive hypometabolism in the PCC, precuneus and lateral temporal lobe, but not the MTL, preceded corresponding cortical thickness degeneration in those with dementia conversion if the FDG-PET was arranged at the MCI stage.

### Electronic supplementary material

Below is the link to the electronic supplementary material.

Supplementary Material 1

Supplementary Material 2

## Data Availability

The datasets generated and/or analysed during the current study are not publicly available due to the nature of this study, but are available from the corresponding author on reasonable request.
