# Supplementary material for: The use of individual-based FDG-PET volume of interest in predicting conversion from mild cognitive impairment to dementia
Source: BMC Med Imaging. 2024 Mar 28;24:75. doi: 10.1186/s12880-024-01256-x (PMC10976703; doi:10.1186/s12880-024-01256-x)
Supplement: Supplementary file 1 — Supplementary Material 1 [file 12880_2024_1256_MOESM1_ESM.docx]

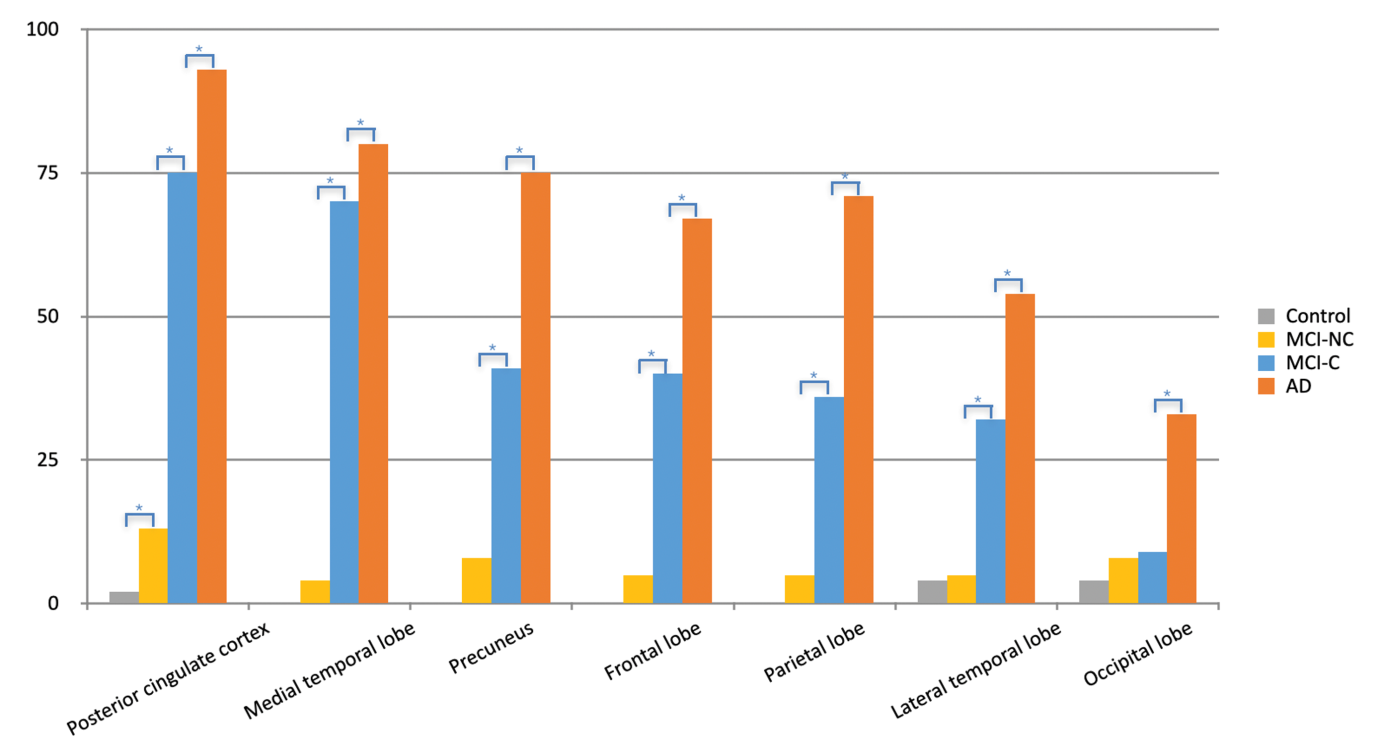


Supplementary Figure 1. Percentages of hypometabolism of seven preselected volume-of-interests in baseline FDG-PET among the four groups. Hypometabolism was defined as having a Z score of < -2. * p < 0.05.

Abbreviations: MCI-NC, non-converter of mild cognitive impairment; MCI-C, converter of mild cognitive impairment; AD, Alzheimer’s disease.


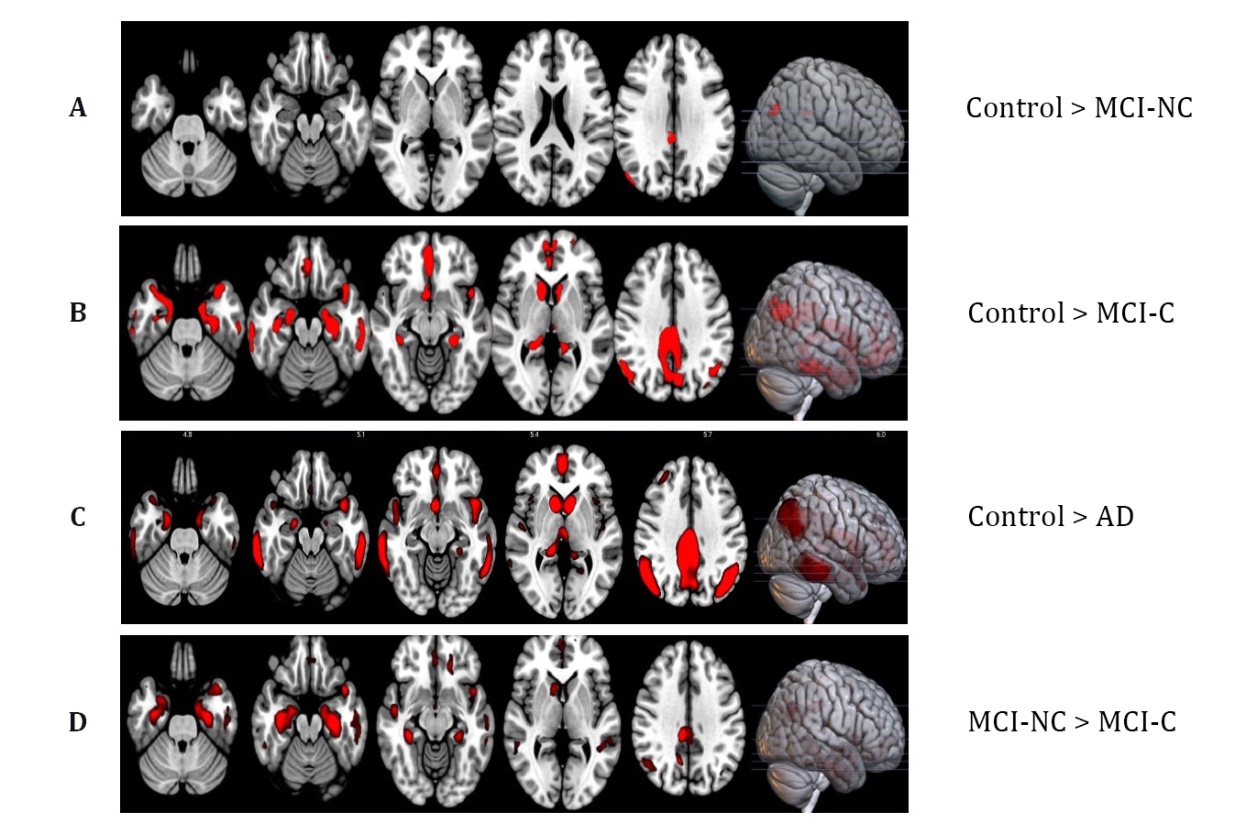


Supplementary Figure 2. Statistical parametric maximum intensity as projected onto a normal MRI set spatially normalized into the Montreal Neurological Institute normal MRI template, showed significant decline of SUVr in the patients with MCI-NC compared with the controls in the PCC (A). Hypometabolism in the precuneus, PCC, MTP, and temporo-parietal cortices was detected in the MCI-C and AD groups compared with the controls (B-C). PCC and MTP hypometabolism was detected in the MCI-NC group compared with the MCI-C group (D). Height threshold is <0.001, corrected for multiple comparisons.

**Supplementary Table 1. Correlations between the baseline FDG-PET Z scores and CASI**

|  | Mental manipulate | Attention | Orientation | Long-term memory | Short-term memory | Abstract thinking | Drawing | Verbal fluency | Language | Total score |
| --- | --- | --- | --- | --- | --- | --- | --- | --- | --- | --- |
| **AD** |  |  |  |  |  |  |  |  |  |  |
| Frontal | .115 | .26 | .294 | .182 | **.436^**^** | .022 | .141 | .**317^*^** | .124 | .261 |
| PCC | **.423^**^** | **.517^**^** | **.622^**^** | **.364^*^** | **.648^**^** | .115 | **.486^**^** | **.464^**^** | **.496^**^** | **.585^**^** |
| Precuneus | **.353^*^** | **.447^**^** | **.473^**^** | **.342^*^** | **.527^**^** | .076 | **.420^**^** | **.432^**^** | **.409^**^** | **.487^**^** |
| Parietal | **.349^*^** | **.540^**^** | **.461^**^** | **.343^*^** | **.466^**^** | .078 | **.469^**^** | **.404^**^** | **.477^**^** | **.497^**^** |
| Occipital | **.388^*^** | **.509^**^** | **.457^**^** | **.365^*^** | **.431^**^** | .058 | **.549^**^** | **.352^*^** | **.512^**^** | **.504^**^** |
| Lat. temporal | **.438^**^** | **.520^**^** | **.534^**^** | **.460^**^** | **.577^**^** | .214 | **.442^**^** | **.470^**^** | **.566^**^** | **.588^**^** |
| Med. temporal | .28 | .217 | **.547^**^** | **.383^*^** | **.549^**^** | .263 | **.428^**^** | .302 | **.466^**^** | **.499^**^** |
| **MCI-C** | | | | | | | | | | |
| Frontal | -.21 | -.036 | -.057 | -.167 | -.013 | -.049 | -.108 | .085 | -.062 | -.103 |
| PCC | .155 | .204 | **.376^*^** | .101 | .225 | .182 | .222 | **.312^*^** | .208 | **.351^*^** |
| Precuneus | .013 | .033 | .073 | .015 | .046 | .06 | .035 | .22 | .151 | .104 |
| Parietal | .007 | .066 | .145 | .107 | .173 | .183 | .129 | .16 | .16 | .191 |
| Occipital | -.003 | -.03 | .083 | .075 | .126 | .093 | .016 | .158 | .098 | .111 |
| Lat. temporal | .037 | .062 | .195 | .154 | .26 | .282 | .092 | .201 | .108 | .245 |
| Med. temporal | -.142 | .001 | .123 | -.033 | **.306^*^** | .177 | -.075 | -.002 | .018 | .085 |
| **MCI-NC** | | | | | | | | | | |
| Frontal | -.227 | -.253 | .178 | -.021 | .271 | -.105 | -.046 | -.001 | -.177 | -.017 |
| PCC | -.004 | -.188 | .163 | .205 | **.449^**^** | .02 | .036 | .129 | -.103 | .173 |
| Precuneus | -.027 | -.162 | .053 | .126 | **.337^*^** | -.041 | .034 | .087 | -.123 | .086 |
| Parietal | -.006 | -.17 | .082 | .125 | .31 | -.089 | .063 | -.014 | -.102 | .071 |
| Occipital | -.061 | -.167 | -.123 | -.021 | .099 | -.136 | .072 | -.151 | -.185 | -.098 |
| Lat. temporal | .018 | -.214 | .076 | .078 | .257 | -.014 | .141 | -.014 | -.235 | .065 |
| Med. temporal | .067 | **-.321^*^** | -.044 | .148 | .168 | .133 | .104 | -.126 | .009 | .052 |
| **Control** | | | | | | | | | | |
| Frontal | .112 | -.185 | .017 | .25 | .054 | -.024 | -.103 | .292 | .289 | .176 |
| PCC | -.109 | -.183 | -.164 | .12 | .009 | .024 | -.056 | .29 | .125 | .048 |
| Precuneus | .05 | -.262 | -.022 | .156 | -.005 | -.088 | .032 | .297 | .179 | .097 |
| Parietal | -.012 | -.249 | .014 | .229 | -.127 | -.223 | -.011 | .328 | .246 | .045 |
| Occipital | .112 | -.246 | .064 | .285 | -.11 | -.227 | .041 | **.402^*^** | .255 | .133 |
| Lat. temporal | .075 | -.174 | -.012 | .311 | .054 | -.155 | -.068 | .307 | .295 | .147 |
| Med. temporal | .218 | -.037 | .153 | .284 | .291 | .159 | -.154 | .32 | .273 | **.368^*^** |

Data represents correlation coefficient, adjusted for years of education. * *p* < 0.05; ** *p* < 0.01.

Abbreviations: MCI-NC, non-converter of mild cognitive impairment; MCI-C, converter of mild cognitive impairment; AD, Alzheimer’s disease; CASI, cognitive ability screening instrument; FDG-PET, fluorodeoxyglucose positron emission tomography; PCC, posterior cingulate cortex; Lat. temporal, lateral temporal; Med. temporal, medial temporal.

**Supplementary Table 2. Comparisons between the two MCI groups in the six consecutive cognitive tests**

| Case numbers | Total (n=82) | MCI-NC (n=38) | MCI-C (n=44) | P value |
| --- | --- | --- | --- | --- |
| Serial age |  |  |  |  |
| age at 1st test | 70.73±7.48 | 69.42±7.46 | 71.86±7.39 | .117 |
| age at 2nd test | 71.73±7.59 | 70.34±7.50 | 72.93±7.55 | .105 |
| age at 3rd test | 72.79±7.74 | 71.24±7.77 | 73.95±7.59 | .115 |
| age at 4th test | 73.68±7.60 | 71.90±7.44 | 74.95±7.54 | .078 |
| age at 5th test | 74.61±7.66 | 72.41±7.20 | 76.17±7.67 | **.033^*^** |
| age at 6th test | 75.89±7.86 | 74.00±7.59 | 77.11±7.89 | .098 |
| Serial MMSE |  |  |  |  |
| MMSE^1^ (n=82) | 22.61±4.06 | 23.50±4.36 | 21.84±3.66 | **.019^*^** |
| MMSE^2^ (n=82) | 22.00±4.18 | 22.71±4.41 | 21.39±3.91 | .116 |
| MMSE^3^ (n=77) | 21.26±4.59 | 22.79±4.19 | 20.11±4.58 | **.003^**^** |
| MMSE^4^ (n=74) | 20.49±5.50 | 22.39±4.77 | 19.12±5.63 | **.006^**^** |
| MMSE^5^ (n=70) | 19.94±5.71 | 22.38±4.62 | 18.22±5.83 | **.001^**^** |
| MMSE^6^ (n=61) | 19.10±6.24 | 21.96±5.98 | 17.24±5.76 | **.003^**^** |
| Difference of MMSE |  |  |  |  |
| MMSE^2^-MMSE^1^(n=82) | -0.61±2.42 | -0.79±2.61 | -0.45±2.26 | .851 |
| MMSE^3^-MMSE^1^ (n=77) | -1.13±2.95 | -0.33±2.90 | -1.73±2.87 | .067 |
| MMSE^4^-MMSE^1^ (n=74) | -1.82±4.21 | -0.68±3.40 | -2.65±4.57 | .117 |
| MMSE^5^-MMSE^1^ (n=70) | -2.37±4.00 | -0.97±2.98 | -3.37±4.35 | **.025^*^** |
| MMSE^6^-MMSE^1^ (n=61) | -3.11±5.03 | -0.83±4.67 | -4.59±4.75 | **.003^**^** |
| Serial CDR |  |  |  |  |
| CDR^1^ (n=82) | 0.52±0.17 | 0.50±0.16 | 0.53±0.17 | .354 |
| CDR^2^ (n=82) | 0.52±0.18 | 0.47±0.20 | 0.56±0.16 | **.043^*^** |
| CDR^3^ (n=77) | 0.56±0.19 | 0.53±0.17 | 0.59±0.20 | .169 |
| CDR^4^ (n=74) | 0.64±0.28 | 0.56±0.21 | 0.70±0.31 | **.048^*^** |
| CDR^5^ (n=70) | 0.70±0.37 | 0.60±0.25 | 0.77±0.42 | .110 |
| CDR^6^ (n=61) | 0.77±0.37 | 0.69±0.36 | 0.82±0.38 | .070 |
| Serial CASI |  |  |  |  |
| CASI^1^ (n=82) | 76.24±13.01 | 77.84±13.91 | 74.85±12.17 | .110 |
| CASI^2^ (n=81) | 74.96±13.23 | 76.22±14.69 | 73.90±11.94 | .350 |
| CASI^3^ (n=75) | 73.77±15.81 | 79.02±14.06 | 69.87±16.05 | **.006^**^** |
| CASI^4^ (n=72) | 69.80±17.70 | 76.02±13.73 | 65.60±18.95 | **.011^*^** |
| CASI^5^ (n=66) | 65.60±20.96 | 75.60±16.64 | 59.11±21.10 | **.001^**^** |
| CASI^6^ (n=59) | 61.98±21.23 | 71.49±18.71 | 56.33±20.83 | **.002^**^** |
| Difference of CASI |  |  |  |  |
| CASI^2^-CASI^1^ (n=81) | -1.21±7.81 | -1.51±9.45 | -0.95±6.21 | .898 |
| CASI^3^-CASI^1^ (n=75) | -1.85±10.11 | 2.08±9.26 | -4.77±9.82 | **.002^**^** |
| CASI^4^-CASI^1^ (n=72) | -6.01±12.55 | -1.51±9.15 | -9.04±13.68 | **.021^*^** |
| CASI^5^-CASI^1^ (n=66) | -9.99±14.51 | -2.08±12.00 | -15.12±13.79 | **<.001^***^** |
| CASI^6^-CASI^1^ (n=59) | -13.66±15.30 | -5.97±13.29 | -18.24±14.72 | **.001^**^** |

Data presented as mean ± standard deviation; Mann-Whitney test for continuous variables (MMSE, CASI), Fisher’s exact test for categorical variables (CDR). Superscript numbers following test score represents the time of measurement. * *p* < 0.05; ** *p* < 0.01; *** *p* < 0.001

Abbreviations: MCI-NC, non-converter of mild cognitive impairment; MCI-C, converter of mild cognitive impairment; MMSE, mini-mental state examination; CDR, clinical dementia rating; CASI: cognitive ability screening instrument total scores.

**Supplementary Table 3. FDG-PET Z scores of two scans between MCI-C and MCI-NC**

| MCI-C compares to MCI-NC | MCI-C (n=24) | MCI-NC (n=13) | *p* value^†^ |
| --- | --- | --- | --- |
| Frontal^1^ | -0.68±0.83 | -0.03±1.03 | .021^*^ |
| Frontal^2^ | -1.04±0.96 | -0.09±1.00 | .005^**^ |
| PCC^1^ | -1.58±0.78 | -0.11±1.16 | <.001^***^ |
| PCC^2^ | -1.96±0.68 | -0.32±1.21 | <.001^***^ |
| Precuneus^1^ | -0.95±0.88 | 0.02±1.18 | <.015^*^ |
| Precuneus^2^ | -1.22±0.99 | -0.13±1.14 | .004^**^ |
| Parietal^1^ | -0.47±1.00 | 0.22±1.12 | .018^*^ |
| Parietal^2^ | -0.80±1.09 | 0.16±1.08 | .013^*^ |
| Occipital^1^ | -0.24±0.93 | 0.29±1.04 | .030^*^ |
| Occipital^2^ | -0.53±1.06 | 0.36±0.87 | .005^**^ |
| Lat. temporal^1^ | -0.84±1.04 | 0.37±0.92 | .002^**^ |
| Lat. temporal^2^ | -1.29±0.97 | 0.29±0.92 | <.001^***^ |
| Med. temporal^1^ | -1.66±0.89 | 0.10±0.91 | <.001^***^ |
| Med. temporal^2^ | -1.94±0.87 | 0.08±1.12 | <.001^***^ |

† using Mann-Whitney U test; Superscript 1 indicates the first scan of FDG-PET; 2 indicates the second scan of FDG-PET. * *p* < 0.05; ** *p* < 0.01; *** *p* < 0.001

Abbreviations: MCI-NC, non-converter of mild cognitive impairment; MCI-C, converter of mild cognitive impairment; PCC, posterior cingulate cortex; Lat. temporal, lateral temporal; Med. temporal, medial temporal.

**Supplementary Table 4. Details of the amyloid and tau PET analysis in the MCI patients**

|  | A-T- | A-T+ | A+T- | A+T+ | P |
| --- | --- | --- | --- | --- | --- |
| Patient Number | 8 | 0 | 0 | 18 |  |
| Age, years | 76.1±6 | NA | NA | 73.3±8 | 0.112 |
| Sex, *n* (%) male | 4 (50%) | 0 | 0 | 8 (44.4%) | 0.801 |
| Education, years | 7.3±3.1 | NA | NA | 5.7±3.5 | 0.01 |
| Duration^*^ , years | 3.8±1.6 | NA | NA | 3.0±1.5 | 0.338 |
| MCI-NC , *n* (%) | 7 (58.3%) | 0 | 0 | 5 (41.7%) |  |
| MCI-C , *n* (%) | 1 (7.1%) | 0 | 0 | 13 (92.9%) |  |

*P* values from two-sided statistical tests are reported.

^*^Duration after FDG-PET.

Abbreviations: MCI-NC, non-converter of mild cognitive impairment; MCI-C, converter of mild cognitive impairment; A, amyloid; T, tau.
