# Supplementary material for: The use of individual-based FDG-PET volume of interest in predicting conversion from mild cognitive impairment to dementia
Source: BMC Med Imaging. 2024 Mar 28;24:75. doi: 10.1186/s12880-024-01256-x (PMC10976703; doi:10.1186/s12880-024-01256-x)
Supplement: Supplementary file 2 — Supplementary Material 2 [file 12880_2024_1256_MOESM2_ESM.docx]

Supplementary file

**Standard protocol approval, registration and patient consent**

We selected controls, patients with MCI due to AD (Albert et al. 2011) and patients with AD dementia (Jack et al. 2018) from a database at our institute. The database contains a longitudinal clinical and MRI cohort focusing on older cognitive-compliant subjects. Patients or controls in the database receive regular follow-up at the department of general neurology. All patients underwent annual cognitive assessments and structural MRI assessments every 1.5-2 years.

**MRI acquisition and preprocessing steps**

In our protocol, 3D high-resolution T1-weighted MRI was acquired at 1.5-2-year intervals, depending on the enrolled protocols. Each subject received a T2-weighted MRI scan to confirm the absence of pathological lesions in the brain. Those with a higher white matter hyperintensity score (Fazekas scale >2) were excluded. 3D T1 MR images were obtained using a 3T GE Discovery 750 (GE Medical Systems, Milwaukee, WI, USA) and acquired using a T1-weighted, inversion-recovery-prepared, three-dimensional, gradient-recalled acquisition in a steady-state sequence [repetition time (TR) = 12.24 msec; echo time (TE) = 5.18 msec; field of view (FOV) = 256 × 256; matrix size= 256 × 256; number of excitations (NEX) = 1; inversion time (TI) = 450 msec; flip angle = 15] with a 1-mm slice sagittal thickness with a resolution of 0.5 × 0.5 × 1 mm^3^.  All MRI scans were preprocessed on the same workstation (Macintosh iMac Pro 2017, macOS Catalina, version 10.15.16) using FreeSurfer image analysis suite v7.1.1 ([http://surfer.nmr.mgh.harvard.edu](http://surfer.nmr.mgh.harvard.edu/)) for cortical parcellation. For each 3D T1 image, we performed both cross-sectional and longitudinal streams, which segmented all serial timepoints at the same time to increase the longitudinal stability.

**Group preprocessing of PET**

We used PETSurfer (<https://surfer.nmr.mgh.harvard.edu/fswiki/PetSurfer>) to register a PET scan to its corresponding time point MRI (Mak et al. 2019). Regional values could be output with or without geometric transfer matrix partial volume correction (PVC). Although PVC of PET images was proposed decades ago, we did not use PVC in this study based on the report of Schwarz et al. (Schwarz et al. 2019). In their report, the effects of PVC in amyloid PET using geometric transfer matrix PVC had worse precision than those without PVC. In addition, a longitudinally stabilized approach did not improve the properties. Since we assessed both cross-sectional and longitudinal analysis using FreeSurfer, which only implemented a geometric transfer matrix PVC, we decided to omit PVC correction in the processing pipeline. PETSurfer has no longitudinal pipeline for analyzing serial PET scans, so we coregistered PET images to the longitudinal stream preprocessed MRI segments and concatenated all longitudinal PET to perform the longitudinal stream pipeline.

Results

**Baseline FDG-PET topography and its relationship with cognitive performance**

The same analysis was conducted with the CASI and its subdomains to clarify whether hypometabolism in different brain regions was associated with cognitive impairment of different domains. As shown in Supplementary Table 1, the extended hypometabolism changes in AD were correlated with generally lower cognitive scores except for abstract thinking. For MCI-C, the Z score of the PCC correlated with orientation, verbal fluency and CASI total scores, while MTL Z scores correlated with short-term memory. For MCI-NC, both PCC and precuneus Z scores correlated with short-term memory. For the control group, MTL correlated with CASI total scores.

**Longitudinal cognitive trajectory**

Data for the cognitive features of the two MCI groups are listed in the Supplementary results and Supplementary Table 2. The average age of the two groups at the first measurement was 70.7 years, and was about one year older at each subsequent measurement. The age of the MCI-C patients was significantly older than MCI-NC the patients at the fifth measurement (76.17 versus 72.41); otherwise, there was no difference in age between the two groups. From the third measurement, both MMSE and CASI scores were significantly lower in the MCI-C group compared with the MCI-NC group, while the changes in CDR were less obvious. The differences in declining trends were more obvious in the CASI than in the MMSE. In the CASI, a significant decline was noted from the third measurement (decrease by 4.77 points compared with the first study in the MCI-C group, while the MCI-NC group showed an increase by 2.08 points compared with the first study). In the MMSE, a significant decline was noted until the fifth measurement (decrease by 3.37 points in the MCI-C group and 0.97 points in the MCI-NC group compared with the first study).

Discussion

**Evolution in FDG-PET reflected cognitive decline**

In contrast to the predictive value of conversion, the cross-sectional FDG-PET topography did not reflect the severity of general cognitive impairment in MCI. This could be related to the ceiling effect of cognitive tests in MCI subjects, especially those evaluating overall cognitive impairment, such as the MMSE, which may not be able to reflect the subtle cognitive impairment in MCI. Indeed, in the analysis of CASI and its subdomains, we found correlations between cross-sectional hypometabolism and cognitive impairment in the MCI subjects (the PCC was correlated with CASI total score, orientation and verbal fluency, and the MTL was correlated with short-term memory). Moreover, the cognitive decline in the subjects with MCI was more variable than in those with AD, with some experiencing less impairment and some remaining stable; meanwhile, the FDG-PET hypometabolism pattern of the subjects with MCI was more sparse than that of those with AD. This may be a limitation of a cross-sectional study. Based on the GEE model, we analyzed the correlations between longitudinal FDG-PET evolutional patterns and cognitive decline. We found that the reduction in FDG-PET uptake in the PCC, precuneus and lateral temporal lobe was correlated with the decline in MMSE and CASI scores (while educational level and male sex were considered to be protective factors). One may wonder why the SUVr change in the MTL was not associated with cognitive decline if it is a predictor of MCI conversion. As mentioned earlier, the hypometabolic change of the MTL may reach a peak in the early stage of MCI; as a result, there was no significant correlation with the ongoing cognitive decline. In fact, hippocampal hypometabolism can be found in normal aging adults with memory complaints who are later diagnosed with MCI or AD (Mosconi et al. 2008). MTL or hippocampal hypometabolism may foreshadow cognitive decline at a stage even earlier than MCI; further studies are needed to investigate this notion.
